# Preconditioning Shields Against Vascular Events in Surgery (SAVES), a multicentre feasibility trial of preconditioning against adverse events in major vascular surgery: study protocol for a randomised control trial

**DOI:** 10.1186/s13063-015-0678-1

**Published:** 2015-04-23

**Authors:** Donagh Healy, Mary Clarke-Moloney, Brendan Gaughan, Siobhan O’Daly, Derek Hausenloy, Faisal Sharif, John Newell, Martin O’Donnell, Pierce Grace, John F Forbes, Walter Cullen, Eamon Kavanagh, Paul Burke, Simon Cross, Joseph Dowdall, Morgan McMonagle, Greg Fulton, Brian J Manning, Elrasheid AH Kheirelseid, Austin Leahy, Daragh Moneley, Peter Naughton, Emily Boyle, Seamus McHugh, Prakash Madhaven, Sean O’Neill, Zenia Martin, Donal Courtney, Muhammed Tubassam, Sherif Sultan, Damian McCartan, Mekki Medani, Stewart Walsh

**Affiliations:** Department of Vascular Surgery, University Hospital Limerick, Saint Nessan’s Road, Dooradoyle, Limerick, Ireland; University of Limerick, Castletroy, Limerick, Ireland; National Cardiovascular and Stroke Research Network, Irish Heart Foundation, 50 Ringsend Road, Dublin, Ireland; The Hatter Cardiovascular Institute, University College London, 67 Chenies Mews, London, WC1E 6HX UK; NUI Galway, University Road, Galway, Ireland; Health Research Board Clinical Research Facility Galway, National University of Ireland, Galway, Geata an Eolais, University Road, Galway, Ireland; Waterford Regional Hospital, Dunmore Road, Waterford, Ireland; Cork University Hospital, Corcaigh, Wilton, Co. Cork, Ireland; Beaumont Hospital, Beaumont Road, Dublin 9, Ireland; Department of Vascular Surgery, Beaumont Hospital, Beaumont Road, Dublin 9, Ireland; St. James’s Hospital, James Street, Dublin 8, Ireland; Galway University Hospital, Newcastle Road, Galway, Ireland; Department of Vascular Surgery, Waterford Regional Hospital, Dunmore Road, Waterford, Ireland

**Keywords:** remote preconditioning, vascular surgery, perioperative complications

## Abstract

**Background:**

Patients undergoing vascular surgery procedures constitute a ‘high-risk’ group. Fatal and disabling perioperative complications are common. Complications arise via multiple aetiological pathways. This mechanistic redundancy limits techniques to reduce complications that target individual mechanisms, for example, anti-platelet agents. Remote ischaemic preconditioning (RIPC) induces a protective phenotype in at-risk tissue, conferring protection against ischaemia-reperfusion injury regardless of the trigger. RIPC is induced by repeated periods of upper limb ischaemia-reperfusion produced using a blood pressure cuff. RIPC confers some protection against cardiac and renal injury during major vascular surgery in proof-of-concept trials. Similar trials suggest benefit during cardiac surgery. Several uncertainties remain in advance of a full-scale trial to evaluate clinical efficacy. We propose a feasibility trial to fully evaluate arm-induced RIPC’s ability to confer protection in major vascular surgery, assess the incidence of a proposed composite primary efficacy endpoint and evaluate the intervention’s acceptability to patients and staff.

**Methods/Design:**

Four hundred major vascular surgery patients in five Irish vascular centres will be randomised (stratified for centre and procedure) to undergo RIPC or not immediately before surgery. RIPC will be induced using a blood pressure cuff with four cycles of 5 minutes of ischaemia followed by 5 minutes of reperfusion immediately before the start of operations. There is no sham intervention. Participants will undergo serum troponin measurements pre-operatively and 1, 2, and 3 days post-operatively. Participants will undergo 12-lead electrocardiograms pre-operatively and on the second post-operative day. Predefined complications within one year of surgery will be recorded. Patient and staff experiences will be explored using qualitative techniques. The primary outcome measure is the proportion of patients who develop elevated serum troponin levels in the first 3 days post-operatively. Secondary outcome measures include length of hospital and critical care stay, unplanned critical care admissions, death, myocardial infarction, stroke, mesenteric ischaemia and need for renal replacement therapy (within 30 days of surgery).

**Discussion:**

RIPC is novel intervention with the potential to significantly improve perioperative outcomes. This trial will provide the first evaluation of RIPC’s ability to reduce adverse clinical events following major vascular surgery.

**Trial Registration:**

www.clinicaltrials.gov NCT02097186 Date Registered: 24 March 2014

## Background

Ischaemic preconditioning is a phenomenon whereby a brief period of nonlethal ischaemia in a tissue renders it resistant to the effects of a subsequent much longer ischaemic insult. It was first described in the canine heart [1]. Subsequent clinical trials showed that ischaemic preconditioning reduced heart muscle damage following coronary artery bypass grafting [2] and liver dysfunction following hepatic resection [3]. Following cardiac surgery, it is associated with a reduction in critical care stay, arrhythmias and inotrope use [4]. However, ischaemic preconditioning requires direct interference with the target tissues’ blood supply, limiting its clinical utility. Further experimental work suggested that brief ischaemia in one tissue, such as the kidneys, could confer protection on distant organs such as the heart [5]. A similar effect was observed after transient skeletal muscle ischaemia [6]. This effect is referred to as ‘preconditioning at a distance’ or ‘remote ischaemic preconditioning’ (RIPC).

Patients requiring major vascular surgery for end-stage vascular disease constitute a high-risk surgical cohort. Perioperative complications such as myocardial infarction, cerebrovascular accident, renal failure and death are common [7,8]. Multiple potential mechanisms may lead to these complications. For example, myocardial injury may result from systemic hypotension leading to reduced flow across a tight coronary artery stenosis or, alternatively, it may arise due to acute occlusion when an unstable plaque ruptures. Most strategies aimed at perioperative risk reduction target a single potential mechanism. Thus, for example, beta-blockade may prevent myocardial injury due to overwork, but cannot prevent acute coronary occlusion. There is a requirement for a simple, effective intervention that protects tissues against injury via multiple different mechanisms. Remote ischaemic preconditioning (RIPC) may be suitable.

### Proof-of-concept trials

To date, there have been five small trials of RIPC in patients undergoing major vascular surgery (Table 1). The results were mixed. An apparent beneficial effect of RIPC on renal function following open abdominal aortic aneurysm (AAA) repair in one trial [9] could not be replicated in a subsequent smaller study specifically designed to evaluate renal injury [10]. However, RIPC did reduce renal injury biomarkers following elective endovascular aneurysm repair (EVAR) [11]. In carotid endarterectomy patients, RIPC had no significant effect on subclinical cerebral injury, as determined by saccadic latency deteriorations [12]. However, this trial was rendered underpowered by a high level of patient withdrawals. More recently, a further trial conducted in patients undergoing open AAA repair reported that RIPC reduced markers of pulmonary and intestinal injury [13]. A number of additional small trials have assessed the potential value of RIPC in adult cardiac surgery, again with mixed results [2,14]. Most of these small phase 1 and 2a trials did not report clinical outcomes in any detail, focusing appropriately on biomarkers. Recently, a pooled analysis of some of these trials reported a significant reduction in post-operative troponin levels with RIPC [15]. Few of these studies reported clinical outcomes for example, death, myocardial infarction etc. Another recent meta-analysis examined the effect of RIPC on major clinical complications among 2,200 patients who underwent major cardiovascular surgery [16]. This review found no significant clinical benefit with RIPC, and the authors highlighted that this was likely to be related to a lack of sufficient sample size along with methodological heterogeneity in the individual studies.

**Table 1:** **Summary of previous trials of remote ischaemic preconditioning (RIPC) in major vascular surgery**

**Trial reference**	**Number randomised**	**Operation type**	**Main findings**
Ali *et al*. Circulation [9]	82	Elective open AAA repair	RIPC produced significant reductions in post-operative troponin levels, myocardial infarctions and serum creatinine levels
Walsh *et al*. Vasc Endovasc Surgery [10]	40	Elective open AAA repair	RIPC had no significant effect on post-operative renal injury biomarkers.
Walsh *et al*. J Endovasc Ther [11]	40	Elective EVAR	RIPC produced significant reductions in post-operative biomarkers of renal injury
Walsh *et al*. Vasc Endovasc Surgery [12]	70	Elective carotid endarterectomy	RIPC had no significant effect on saccadic latency deteriorations or biomarkers of cardiac injury
Li *et al*. Anesthesiology [13]	62	Elective open AAA repair	RIPC produced significant reductions in biomarkers of intestinal and pulmonary injury

Legend: Table summarising previous trials of remote ischaemic preconditioning in major vascular surgery.

AAA – abdominal aortic aneurysm; EVAR – endovascular aneurysm repair; RIPC – remote ischaemic preconditioning.

At present, although exploratory studies are attractive, there is a notable lack of data on the effect of RIPC on clinical outcomes. Until hard data on clinical outcomes are available, RIPC cannot achieve a place in routine clinical practice.

### Rationale

It is pertinent to continue to investigate RIPC in the setting of major vascular surgery for several reasons. As mentioned earlier, perioperative risk is high in this group of patients and as such it is essential that risk reduction strategies be developed and maximised. Myocardial conditioning with RIPC seems especially attractive given the encouraging preclinical and proof of concept studies and given its benign risk profile. In contrast, other perioperative risk reduction strategies have limitations [17]: preoperative cardiac risk assessment is theoretically attractive but hard data on its efficacy are lacking [18]; pharmacological cardioprotection has largely been disappointing [19] with the exception of beta blockade in high risk groups [20]; prophylactic coronary revascularisation was shown to be often ineffective [21-24] and is only occasionally recommended [25,26]. Notably, the high perioperative morbidity rates in vascular surgery also confer a major practical advantage - as event rates are high, studies are likely to achieve adequate power with smaller sample sizes than would be necessary in lower risk groups.

Ideally, a clinical trial of RIPC in major vascular surgery would be powered to detect a significant reduction in ‘hard’ clinical endpoints (death, myocardial infarction etc.). A primary composite end-point (death, myocardial infarction, cerebrovascular accident, mesenteric ischaemia, and renal failure requiring renal replacement therapy) is proposed as the primary efficacy end-point for a phase 3 trial. Some pilot data from 40 patients randomised at University Hospital Limerick suggest that such an endpoint would occur in about 15% of patients. Assuming that 15% develop this endpoint, a minimum of 726 patients would be required in each arm to demonstrate a reduction to 10% (alpha 0.05, beta 0.8).

The proposed work builds on the previous trials conducted by the chief investigator in this area, a programme consistent with the *‘MRC Framework for the Design and Evaluation of Complex Interventions to Improve Health’*, which suggests core phases to the development of health interventions. This proposal describes the development and piloting phases of the MRC framework. Conducting a full-scale RCT, and economic evaluation, of remote preconditioning ‘standard care’ in this population is likely to require at least 1,500 participants and to be costly. As there are uncertainties regarding rates of eligibility, consent, participation in the intervention and retention for follow-up - and regarding the feasibility/acceptability of the intervention - this feasibility study is essential to inform the design and conduct of a larger scale study.

### Aims and objectives

This feasibility trial aims to address a number of key design uncertainties:

1. With the exception of Li *et al*. [13], the previous vascular trials used the patient’s leg as the preconditioning stimulus while the cardiac trials almost always used the patient’s arm. The arm is preferable due to the much lower incidence of upper limb peripheral vascular disease. The use of arm RIPC in major vascular surgery patients should be assessed using a surrogate marker of efficacy in advance of a full-scale trial.
2. Given the mixed results in the first four trials in major vascular surgery, a phase 2B trial to further evaluate potential efficacy using a surrogate marker is required to justify phase 3 work. This will also provide an accurate estimate of the likely effect size that can be expected in any phase 3 trial.
3. Clinical trial capability in Ireland is underdeveloped relative to other Western countries. An examination of the feasibility of a large multicentre surgical trial particularly with respect to recruitment, drop-off rates, compliance, data collection and randomisation would be beneficial in advance of proceeding with a phase 3 trial.
4. In order to evaluate the efficacy of RIPC in routine clinical practice in a reasonable timeframe at reasonable cost, we intend to use a composite primary efficacy endpoint in any full trial. Data regarding the incidence of this endpoint in major vascular surgery patients are lacking. This feasibility trial will provide robust data regarding this composite endpoint upon which to base power calculations for a phase 3 trial, should it appear justified from evaluation of the surrogate efficacy marker.

The single main research question for the feasibility trial, in terms of PICO (Population; Intervention; Comparator; Outcome) is as follows: ‘In adult patients undergoing elective major vascular surgery, does RIPC induced by brief arm ischaemia and reperfusion, when compared to control, reduce troponin leakage in the first 3 days post-operatively?’. Whilst powered to address this specific research question, this phase 2b trial will also provide insight into the feasibility of running a larger, phase 3 study, will provide data regarding the proposed composite primary efficacy endpoint and will evaluate the use of arm-generated as opposed to leg-generated RIPC in major vascular surgery patients.

## Methods/Design

### Study design

**Figure 1:**
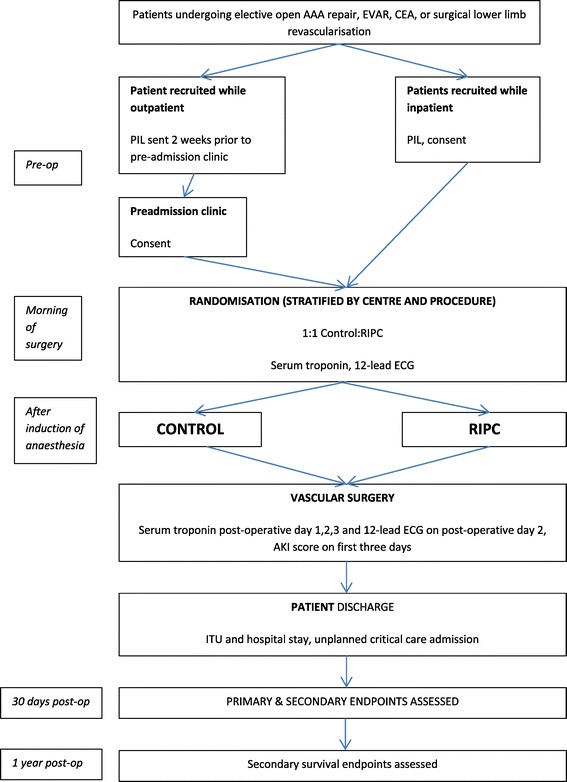
Participant flow through the trial. AAA – abdominal aortic aneurysm; CEA – carotid endarterectomy; ECG –electrocardiography; EVAR – endovascular abdominal aneurysm repair; PIL – patient information leaflet; RIPC – remote ischaemic preconditioning.

This is a randomised controlled pilot trial of the effect of RIPC induced by brief arm ischaemia and reperfusion on troponin levels in the first 3 days post-operatively in patients undergoing elective major vascular surgery. Patients are randomised to receive RIPC or act as controls. There is no sham intervention. The primary study endpoint is determined at 3 days post-operatively. Secondary study endpoints are evaluated at 30 days and one year post-operatively. Participant flow through the trial is described in Figure 1.

### Study setting

Patients undergoing scheduled major vascular surgery will be screened for eligibility in five vascular surgery units in the Republic of Ireland. Patients will receive routine perioperative care according to the current standard of care. During the study period, patients will be discharged home or to continuing care facilities within the geographic area.

### Participants

Patients undergoing elective carotid endarterectomy, open AAA repair, EVAR or surgical lower limb revascularisation (suprainguinal or infrainguinal) will be eligible for inclusion. Patients will be excluded if any of the following apply:

1. Pregnancy
2. Significant upper limb peripheral arterial disease
3. Previous history of upper limb deep vein thrombosis
4. Patients on glibenclamide or nicorandil (these medications may interfere with RIPC)
5. Patients with an estimated pre-operative glomerular filtration rate <30 mls/min/1.73 m^2^
6. Patients with a known history of myocarditis, pericarditis or amyloidosis
7. Patients undergoing fenestrated or branched EVAR.
8. Patients with severe hepatic disease defined as an international normalised ratio >2 in the absence of systemic anticoagulation
9. Patients with severe respiratory disease (for the trial, defined as patients requiring home oxygen therapy)
10. Patients previously enrolled in the trial representing for a further procedure
11. Patients with prior axillary surgery who are thus unsuitable for arm cuff inflation

### Informed consent and ethical approval

In each centre, potentially eligible patients will be selected from two patient groups: (1) outpatients awaiting major vascular surgery or (2) inpatients awaiting major vascular surgery. The process of obtaining informed consent will be conducted in compliance with the principals of good clinical practice and requirements of the approving research ethics committee and other regulatory requirements as appropriate. Consent to enter the study must be sought from each subject only after a full explanation has been given and time allowed for consideration. Signed subject consent will be obtained and a copy given to the subject. It is a right of the subject to refuse to participate or to withdraw at any time from the protocol without giving reasons and without prejudicing treatment. The trial has been reviewed and received approval from the Research Ethics Committees at University Hospital Limerick (final approval 1 May 2014), Cork University Hospital (ECM3 (j) 06/05/14), Waterford University Hospital (final approval 29 July 2014), St. James’s Hospital (2014-01List/05/19-02/2014-07 List25 (3), Beaumont Hospital (REC ref (14/01) and Galway University Hospital (Ref 22/12).

### Randomization, blinding and treatment allocation

Randomisation for the trial will be undertaken using a web-based randomisation system administered by the Health Research Board Clinical Research Facility Galway (HRBCRFG). The use of third-party randomisation will maintain allocation concealment. A unique trial number will be assigned at the time of randomisation. On the morning of surgery, patients will be randomised to one of two groups: either to RIPC or to the control arm. Randomisation will be stratified by centre using randomly selected blocks of 4, 6 and 8. In addition, each centre will be stratified by procedure. The randomisation will be accessed by a designated member of the research team at each site who will not be involved with data collection.

### Intervention

The intervention being tested is a simple intervention that can be undertaken using existing equipment. Immediately before the start of the operation, patients randomised to the RIPC arm will have a standard, CE-approved tourniquet cuff placed around one upper limb. It will then be inflated to a pressure of 200 mmHg for 5 minutes. For patients with a systolic blood pressure >185 mmHg, the cuff will be inflated to at least 15 mmHg above the patient’s systolic blood pressure. The cuff will then be deflated and the arm allowed to reperfuse for 5 minutes. This will be repeated so that each patient receives a total of four ischaemia-reperfusion cycles. There is no sham intervention for those in the control arm.

Previous trials of RIPC in cardiac surgery and percutaneous coronary interventions have generally used the arm as the source of the RIPC stimulus. Four previous trials of RIPC in major vascular surgery (Table 1) used the patients’ legs to generate the stimulus. In these trials, four patients in the RIPC arm developed post-operative critical lower limb ischaemia requiring intervention. The presence of known peripheral arterial disease also precluded the enrolment of about 20% of otherwise eligible patients. Given the success of upper limb RIPC in the cardiac and percutaneous coronary intervention (PCI) trials, the upper limb has been selected as the source for the RIPC stimulus in Preconditioning SAVES. In common with the ERICCA trialists [27], four cycles has been chosen in order to maximise the RIPC stimulus obtained and counteract three potential confounding issues: (i) the lower skeletal muscle bulk in the arm compared to the leg, (ii) the potential resistance to RIPC in aging and diabetic patients and (iii) the effect of variable volatile anaesthetic use.

There is a theoretical possibility that the RIPC stimulus obtained from the upper limb will be insufficient to produce a detectable effect due to the smaller skeletal muscle bulk compared to the lower limb. An adaptive trial design is therefore proposed, in which an interim analysis will be conducted after randomisation of 200 patients. If there is no evidence of potential benefit at this point, the stimulus will be adjusted to include three cycles of 5 minutes lower limb ischaemia alternating with 5 minutes reperfusion. The first lower limb ischaemic episode will commence as soon as the first upper limb ischemic period ends, so that both limbs are being preconditioned in parallel and no additional delay is introduced. If this augmented stimulus is introduced, bilateral ankle-brachial pressure indices <0.8 will be added to the exclusion criteria. The decision to amend the protocol will be made by the Trial Management Committee following advice from the Data Monitoring Committee.

The trial will be undertaken at multiple sites. It is intended to be a pragmatic clinical trial aiming to assess the potential adjunctive value of RIPC in routine clinical practice. Dedicated trial personnel will not be available on all the sites for each patients operation. Consequently, the SAVES trialists have elected not to include a sham intervention in the protocol. To do so would render the trial impractical at many sites.

### Study parameters

#### Baseline data

Patients will all have a full medical history taken and clinical examination as part of their usual care. The following will be recorded:

1. Weight
2. Height
3. Blood pressure
4. Heart rate
5. ECG
6. Gender
7. Ethnicity
8. Date of birth
9. Diabetes mellitus
10. Hypercholesterolaemia
11. Hypertension
12. Previous myocardial infarction
13. Previous coronary revascularisation
14. Previous stroke
15. Atrial fibrillation
16. Peripheral arterial disease
17. Smoking history
18. NYHA class
19. Medication at time of consent (aspirin, clopidogrel, beta-blocker, calcium-channel antagonist, nitrates, cholesterol-lowering agent, ACE inhibitor/A2 receptor antagonist, insulin, metformin, sulphonylurea, or warfarin)
20. Serum creatinine and troponin.

### Perioperative troponin

This will be assessed by measuring serum high-sensitivity troponin I pre-operatively and at 24, 48 and 72 hours post-operatively (four samples per patient). The samples will be analysed in the local laboratory at each site, using local protocols.

### Perioperative 12-lead electrocardiography

A 12-lead ECG is performed routinely as part of the pre-operative assessment for major vascular surgery. This will be used as the trial baseline ECG. Every patient will undergo a second 12-lead ECG on the second post-operative day. The pre- and post-operative ECGs together with the serum troponin levels will be reviewed by the trial cardiologists to determine whether a perioperative silent myocardial infarction has occurred. The reviewing cardiologists will be blinded to trial allocation.

### Length of hospital/intensive therapy unit stay

The duration of hospital stay and intensive therapy unit (ITU) stay have a major impact on health service resource utilisation, and are factors that can be influenced by surgery.

### Acute kidney injury score

The acute kidney injury (AKI) score will be calculated over the first three perioperative days. Creatinine will be measured daily as part of routine care. Urine volumes will be calculated from the fluid balance charts maintained as part of usual care.

### Perioperative data

On the morning of surgery, the patient will be randomised to either the RIPC or control arm using a web-based system. The following information on surgery will be recorded for all patients: anaesthetic induction agents, anaesthetic maintenance, duration of anaesthesia, type of surgery, blood loss, maximum intra-operative heart rate, minimum intra-operative systolic blood pressure. For open AAA patients, the following additional data will be recorded: infra-renal or suprarenal aortic clamp, cross-clamp time, and mannitol use. For carotid endarterectomy patients, shunt use and patch use will be recorded. For endovascular aneurysm repair patients, screening time and total contrast dose will be recorded.

### Data at discharge

At discharge, duration of hospital stay and ITU stay will be recorded.

### Data at 1 year

General practitioners will be contacted 1 year following randomisation to ascertain whether participants have died or suffered myocardial infarction or cerebrovascular accident. The national Hospital In-Patient Episode (HIPE) database will be interrogated for hospital admissions and patient status in the year since surgery.

### Trial outcome measures

#### Primary efficacy endpoint

The trial is intended to pragmatically evaluate the potential of RIPC to improve clinical outcomes among patients undergoing major vascular surgery in routine clinical practice. For the pilot trial, a surrogate marker of efficacy will be used, namely serum troponin I levels. The primary efficacy outcome will be a comparison of the proportion of patients in each arm of the trial who develop a raised serum troponin level in the first three post-operative days. For patients with a baseline troponin level within the normal range, this outcome will be considered positive when any subsequent troponin level at 24, 48 or 72 hours post-operatively is above the upper limit of normal. For patients with a baseline troponin level that is above the normal range, this outcome will be considered positive when any subsequent troponin level at 24, 48 or 72 hours post-operatively is above the baseline value. This is a dichotomous outcome for individual patients.

#### Primary safety endpoint

The intervention under investigation carries a theoretical risk of causing acute upper limb ischaemia or an upper limb deep vein thrombosis. Such an event has not been reported by any of the phase 1 and 2 trials of RIPC in cardiac patients. In addition, there is a theoretical risk of minor adverse events such as upper limb bruising. The primary safety endpoint will again be a composite of major life- or limb-threatening adverse events that could be attributed to the preconditioning stimulus. The components are:

1. Acute upper limb ischaemia
2. Acute upper limb deep vein thrombosis

The components of the primary safety endpoint are defined as follows:

1. Acute upper limb ischaemia, which is defined as the development of ischaemia in the arm used for the preconditioning stimulus requiring systemic anti-coagulation, radiological intervention or surgical intervention; and
2. Acute upper limb deep vein thrombosis, which is defined as the development of thrombus within the subclavian, axillary or brachial vein confirmed in duplex ultrasound and in the same arm as used for the RIPC stimulus.

### Secondary endpoints

Secondary endpoints are identified as follows:

1. The proposed composite primary efficacy endpoint for a phase 3 trial (details below)
2. A comparison of the area under the curve of serial troponin values between the two groups
3. Duration of post-operative hospital stay in days from the date of surgery until the date of discharge to the community
4. Duration of intensive care unit stay in days from the date of surgery
5. Unplanned critical care unit admission defined as any admission to a high dependency or intensive care unit, which was not booked in advance of the date of operation
6. Other post-operative complications (Table 2)
7. Acute kidney injury score in first three perioperative days (Table 3)
8. Death within one year of surgery as determined by contacting the patient’s general practitioner
9. Major adverse cardiac or cerebral event (myocardial infarction, cardiac death, cerebrovascular accident) within 1 year of surgery as determined by contacting the patient’s general practitioner

**Table 2:** **Definitions of other post-operative complications**

Respiratory failure	Unplanned initiation of assisted ventilation (CPAP or IPPV)
Transient ischaemic attack	New onset neurological deficit with lateralising signs, which resolves within 24 hours without evidence of cerebral bleeding or infarction on imaging and confirmed by a stroke physician or neurologist
Vascular graft occlusion	Occlusion of prosthetic or vein graft confirmed by imaging within 30 days of surgery
Wound infection	Erythema, purulent discharge, pyrexia, positive pathogenic organism on culture
Chest infection	Cough, dirty sputum, pyrexia, positive sputum culture +/−pathogenic organism on sputum culture
Urine infection	Pyrexia, leucocytosis, pathogenic organism on urine culture
Abdominal infection	Intra-abdominal abscess formation confirmed on CT scan or at laparotomy
Other sepsis	Sepsis due to any cause other than those listed above for example, line infection
Prolonged ileus	Absence of bowel function requiring the initiation of nutritional support
Pulmonary embolus	Confirmed radiologically or at autopsy
Deep vein thrombosis	Confirmed radiologically or at autopsy
Limb ischaemia	Compromised circulation in a limb requiring a revascularisation procedure within 30 days of surgery
Major limb amputation	Amputation of a limb due to unsalvageable critical ischaemia within 30 days of surgery
Renal infarction	New renal infarction on post-operative imaging
Renal impairment	Post-operative ≥20% increase in serum creatinine from pre-operative baseline

CPAP – continuous positive airway pressure; IPPV – intermittent positive pressure ventilation.

**Table 3:** **Acute kidney injury criteria**

**AKI grade**	**Creatinine criteria**	**Urine output criteria**
1	Rise > 26.4 μmol/L or 150 to 200% of baseline	<0.5 ml/kg/h for >6 h
2	Rise of 200 to 300% of baseline	<0.5 ml/kg/h for >12 h
3	Increase of 300% or creatinine >354 μmol/L with acute rise of at least 44 μmol/L	<0.3 ml/kg/h for >24 h or anuria for 12 h

Legend: Table summarising acute kidney injury criteria.

AKI – acute kidney injury.

### Proposed composite Primary Efficacy Endpoint for Phase 3

The proposed primary efficacy endpoint is a composite Major Adverse Clinical Event (MACE). The selected endpoints represent common, life-threatening complications of major vascular surgery. Ischaemia-reperfusion injury plays a significant role in these events, and thus, it is biologically plausible that RIPC would reduce the frequency of these events. The components are all strictly defined. The occurrence of any of these events in a patient will be classified as a MACE. The occurrence of multiple events in a single patient will also be classified as a single MACE for the purposes of trial analysis. The components of the primary efficacy endpoint are as follows:

1. Cardiovascular death
2. Myocardial infarction
3. Cerebrovascular accident
4. Renal failure requiring renal replacement therapy
5. Mesenteric ischaemia requiring intervention or biopsy-proven ischaemic colitis

These components of the proposed primary efficacy endpoint have been defined as follows:

1. Cardiovascular death, which is defined as death due to a known cardiovascular cause or where the cause of death is unknown that is, no other cause of death is identified either from the medical history or at post-mortem examination.
2. Myocardial infarction, which will include both perioperative myocardial infarction and myocardial infarction following vascular surgery. It will include silent myocardial infarctions as determined by the trial cardiologist upon examination of trial ECGs and troponin results. The diagnosis of a myocardial infarction will require the presence of at least two of the following:
  - Characteristic ischaemic symptoms lasting at least 20 minutes
  - Electrocardiographic changes including acute ST elevation followed by the appearance of Q waves or the loss of R waves, the development of new left bundle branch block, new persistent T wave inversion lasting at least 24 hours or new ST segment depression persisting over 24 hours
  - Positive troponin T (>0.1 ng/ml) or troponin I (>0.1 mg/ml) levels with a characteristic rise and fall in levels
  - New-onset arrhythmia (ventricular or supraventricular tachycardia or fibrillation) with an associated rise in troponin levels
  - Alternatively, myocardial infarction will be recorded if the patient develops sudden unexpected cardiac death involving cardiac arrest with symptoms suggestive of myocardial ischaemia and accompanied by presumably new ST elevation or new left bundle branch block and/or fresh thrombus on coronary angiography and/or at post-mortem, but death occurring before blood samples could be obtained or at a time before the appearance of cardiac troponin I or T in the blood.
3. Cerebrovascular accident, which is defined as new onset neurological deficit, accompanied by evidence of cerebral infarction or intra-cerebral haemorrhage on CT scan, or confirmed at autopsy.
4. Renal failure requiring renal replacement therapy, which is defined as the initiation of any form of renal replacement therapy for any reason within 30 days of major vascular surgery.
5. Mesenteric ischaemia requiring intervention or biopsy-proven ischaemic colitis, which is defined as small or large bowel ischaemia at laparotomy or found at autopsy or histologically proven on biopsy.

### Trial outcome assessment

The predefined outcomes will be recorded in an electronic case record form developed by the HRBCRFG.

### Evaluation of perioperative myocardial infarction

Evaluation of pre- and post-operative electrocardiograms and troponin levels for evidence of perioperative silent myocardial infarction will be undertaken in batches by the trial cardiologist, Dr Faisal Sharif. Dr Sharif will be blinded to the trial allocation of each participant.

### Evaluation of other outcomes

The development of the remaining outcomes will be determined by a trial research nurse in the Dublin site and by research fellows in the remaining three non-Dublin sites. The individuals assessing these primary and secondary outcomes will be blinded to the trial allocation of the patients, in order to minimise potential sources of bias.

### Evaluation of acceptability and barriers to implementation

Whilst RIPC is simple to induce and inexpensive, it does add to the burden of clinical staff in advance of major vascular surgery. Perceptions that the intervention is onerous will significantly impede adoption into routine practice should a clinical benefit be demonstrated. It may also significantly hinder recruitment in any phase 3 trial, as reluctance to complete the preconditioning protocol may result in numerous protocol breaches in the intervention arm. For patients, particularly those undergoing regional anaesthesia rather than general, the intervention may be burdensome and uncomfortable, again negatively impacting upon likely adoption into routine practice.

In order to explore these potential issues in advance of any full-scale trial, the feasibility trial will include a qualitative evaluation of acceptability to both patients and clinical staff together with a qualitative evaluation of any perceived barriers to implementation. These insights may be of value in the subsequent design of a full-scale trial and may help adoption of RIPC in routine practice should the intervention prove clinically and economically-efficacious.

Healthcare professionals at participating practices will be asked to complete a self-administered questionnaire at the end of the study period. The questionnaire will elicit data on profession and practice details, their perceived experience of trial involvement, and open-ended questions to elicit information regarding attitudes to trial involvement, willingness to recruit participants, difficulties that arose during the trial and potential barriers to further research or routine clinical use of the trial intervention.

A convenience sample of 40 patients (10 from each site) will be contacted following their surgery to conduct a brief interview. This will again involve the use of open-ended questions in order to elicit information regarding patients’ attitudes towards the intervention and perceived barriers to implementation.

### Sample size

Currently, the projected sample size for a full phase 3 trial using the composite clinical endpoint mentioned above is 1,500 patients in total. This would provide sufficient power to detect a reduction in the proportion of patients sustaining the endpoint from 15% to 10% (alpha 0.05, beta 0.8). The assumed 15% figure in the control arm is based upon data from a sample of only 40 patients randomised at University Hospital Limerick. Before proceeding with a full-scale trial, and in accordance with the MRC framework for the evaluation of complex interventions, it would be preferable to (i) explore the feasibility of randomising a significant number of surgical patients in the Irish healthcare system context, (ii) undertake further work using a surrogate marker of efficacy to more robustly evaluate the apparent protective effect of RIPC in major vascular patients, (iii) use such a surrogate marker to evaluate the likely effect size of RIPC, and (iv) confirm that arm-induced RIPC is protective in major vascular patients.

In order to fully evaluate objectives (ii) to (iv) above, the feasibility trial design utilises troponin-positive events as the primary outcome. The sample size calculation for the pilot trial is based upon recently published data regarding post-operative troponin-positive events following major vascular surgery. In a cohort of 337 patients undergoing a mix of major vascular procedures (open and endovascular aneurysm repair, carotid endarterectomy or surgical lower limb revascularisation), 135 (40%) developed a post-operative troponin level in excess of the upper limit of normal. At one year post-surgery, patients with a troponin positive event, that is, those with a level in excess of the upper limit of normal, were more likely to have died [28].

The planned patient cohort for this feasibility trial is very similar to that reported above. Moreover, analysis of preliminary data from 40 patients randomised at University Hospital Limerick found that 19/40 patients developed a troponin-positive event (47.5%). Assuming a 40% incidence of troponin-positive events, 166 patients would be required in each arm to demonstrate a reduction to 25% (alpha 0.05, beta 0.8). To account for dropouts and losses to follow-up, we plan to recruit 200 patients to each arm of the trial. This sample will also provide robust data regarding the likely incidence of the proposed composite primary efficacy endpoint for a phase 3 trial. At 25% of the proposed full trial recruitment, the investigators feel it is sufficiently large to allow adequate testing of trial procedures and compliance at multiple sites in line with objective (i) above.

### Statistical analysis plan

The statistical analysis plan has been developed in conjunction with the HRBCRF Galway. The statisticians will be blinded to the trial allocation for all analyses, with the two trial arms being labelled A and B.

### Interim analysis

An interim analysis will be undertaken once 200 patients have been recruited and 30-day follow-up accrued. This analysis will be undertaken by Dr John Newell, HRBCRF Galway. The interim analysis will be reviewed by the Data Monitoring Committee (Dr John Newell, Prof Martin O’Donnell, and Professor Walter Cullen) and used to provide a recommendation to the Trial Management Committee as to whether the adaptive clauses in the trial design should be invoked.

### Final analysis

Once the trial has closed, a final analysis of the data will be conducted by Dr Newell, the trial biostatistician. The trial arms will be labelled A and B, to maintain blinding. Baseline characteristics will be compared between the trial arms to evaluate the efficacy of randomisation. The proportions of patients with troponin positive events, components of the composite clinical outcome, primary safety endpoints, troponin levels, unplanned critical care admission and other post-operative complications will be compared between the trial arms. Hospital stay and intensive care unit stay will also be compared between the two trial arms. One-year survival and cardiac- and cerebral-event free survival will be compared. These results will all be presented in the final trial report.

### Subgroup analyses

Experimental data suggest that the protective effect of RIPC may be attenuated in diabetic patients. This trial provides an opportunity to evaluate the relationship between RIPC and diabetes in clinical practice. There will be two components to this analysis. In the diabetic subgroup, the proportion of patients suffering a troponin-positive event will be compared between those patients who receive RIPC and patients who are controls. In addition, the proportion of patients suffering a troponin-positive event will be compared between diabetic RIPC patients and non-diabetic RIPC patients, in order to determine whether the effect size of RIPC differs significantly in diabetic patients. These data will inform subsequent design of a phase 3 trial, should it appear justified.

There have also been suggestions that RIPC is attenuated in older patients. Again, this trial provides an opportunity to investigate this potential relationship further. Proportions of the patients sustaining a troponin-positive event will be compared between patients aged ≥80 years at randomisation and patients aged <80 years at randomisation. The proportion with a troponin positive event will also be compared between RIPC and control patients in the ≥80 subgroup. A between group analysis will compare the proportions of troponin positive events between RIPC patients in the ≥80 and <80 groups. These data will again inform subsequent design of a phase 3 trial, should it appear justified.

### Health economics

Within trial economic analysis of direct resource costs and health outcomes will be conducted on an intention to treat basis. A health service perspective will be adopted for measuring and valuing health care utilisation. Using a case-mix approach, we will estimate acute hospital one-year cumulative costs based on the index episode and subsequent events defined in the MACE composite clinical endpoint. No discounting of direct resource costs will be conducted as the time horizon will be limited to one year for within trial analysis.

Survival times will be adjusted using preference or utility weights assigned to each of the events in the MACE to calculate quality adjusted life years (QALYs). The primary treatment effect in the economic analysis will be estimated using an individual level regression model for average (mean) incremental costs and incremental QALYs. The model will consider the joint distribution of costs and QALYs using a general specification that will allow for different parametric and conditional distributions. We also plan to use a Bayesian model with minimally informative priors for means and large variances, following the general framework outlined by Baio [29]. Model parameter uncertainty will be addressed using probabilistic sensitivity analysis summarised using the cost-effectiveness acceptability curve. The heterogeneity of economic treatment effects will be assessed using pre-defined strata on age and people with diabetes.

Longer run modelling will estimate the distribution of costs and QALYs calculated over the expected patient lifetimes. A microsimulation model will be calibrated using information gained from the within trial analysis of cost-effectiveness combined with additional data from (i) trials and observational studies reporting longer run costs, survival and health related quality of life following RIPC and (ii) expert beliefs on the distributions of parameters where information is less readily available. The structural uncertainty in the long run model will be addressed using model averaging methods [30].

### Qualitative data analysis

Each interview will be transcribed verbatim, following which, the transcript will be reviewed by the researchers for accuracy. Each interview will be ‘openly coded’ (to allow development of categories of concepts, without making any prior assumptions). The researcher will analyse half the interviews first to identify common codes for each group of respondents. These coded transcripts will be reviewed by a second researcher to ensure the interviews are coded correctly and consistently and the codes identified by the researcher were the same as those identified by the expert. The second half of the transcripts will be consequently added to the analysis one-by-one allowing for the monitoring of data saturation.

For the purpose of this research, thematic analysis will be used to analyse qualitative data. This approach has many benefits for studies such as this, which are interpretive in nature, as it is a ‘method for identifying, analysing and reporting patterns (themes) within data’ [31]. The process of thematic analysis is concerned with the basic to advanced encoding of data, which are subsequently developed to themes. Themes are described as ‘patterns found in the information’ that can be developed in an inductive manner from raw information but also deductively from prior/existing research and knowledge. This flexible approach can also be seen in how themes identified at one level can help the researcher describe their observations and at a more advanced level allow the researcher to interpret aspects of the phenomenon under study [31].

Qualitative data analysis will be systematic and organised in order to easily locate information within the data set when tracing results, providing examples in context [31]. The qualitative research software Nvivo v8 will be used to facilitate the coding of this data. Such research software allows the researcher to manage, shape and make sense of unstructured information; it also facilitates the complex organisation and retrieval of data (www.qsrinternational.com). The analysis will follow a ‘5-Step Analysis’ approach whereby data is reviewed, examined, coded, themes generated and defined [31]. To achieve validity in the coding/analysis of data, two reviewers will code data independently and inter-rater reliability measures will be computed based on this coding. Coding consistency will be maintained throughout the coding process and will be reviewed by regular meetings between researchers and principal investigator. The findings will be compared with other study findings (validity and credibility). The researchers will present the findings to participants to determine if the study findings reflect their experience of the topic under study (member checking). Illustrative quotes will be used to emphasise points made by the participants.

### Dissemination plan

It is the intention of the trial team to disseminate the results of the trial as widely as possible. This is likely to be through publication in a peer-reviewed vascular surgery journal, and also via presentations at National and International Cardiology Conferences. Publication will be in accordance with the CONSORT guidelines. The trial will be registered before randomisation commences. The results will be submitted for presentation at major vascular surgery conferences. It is anticipated that the trial manuscript will be submitted for publication by 2016. The paper will be attributed to the Preconditioning SAVES Group. Individual clinicians involved in the trial will be listed by centre at the end of the trial.

## Discussion

### Strengths

This is the first trial seeking to evaluate the role of remote preconditioning as a protective adjunct in a typical population of elective major vascular surgery patients. It will have sufficient power to detect a reduction in post-operative troponin-positive events, which are known to adversely affect survival following major vascular surgery. The inclusion of a qualitative evaluation of staff and patient experiences with RIPC will also provide formal data on the intervention’s acceptability. The adaptive design allows for evaluation of a combined arm/leg RIPC stimulus should it appear that the arm stimulus alone is insufficient at the interim analysis stage.

### Weaknesses

Ideally, patients randomized to the control arm would receive a sham intervention. Trial staffing is minimal. In the absence of dedicated trial staff at each site to undertake the intervention, the inclusion of a sham intervention was felt to be excessively onerous and likely to adversely impact on trial participation and enrolment by the surgical teams.

## Trial status

Recruitment for the trial began 1 June 2014.

## Abbreviations

AAA: abdominal aortic aneurysm
ACE: angiotensin-converting enzyme
AKI: acute kidney injury
CPAP: continuous positive airway pressure
CT: computed topography
ECG: electrocardiography
EVAR: endovascular aneurysm repair
HRBCRFG: Health Research Board Clinical Research Facility Galway
IPPV: intermittent positive pressure ventilation
ITU: Intensive Therapy Unit
MACE: major adverse clinical event.
NYHA: New York Heart Association
PICO: population, intervention, comparator, outcome
PCI: percutaneous coronary intervention
QALY: quality-adjusted life years
RCT: randomised control trial
RIPC: remote ischaemic preconditioning
